# Impact of Genetic Variants in Estrogen Receptor-β Gene in the Etiology of Uterine Leiomyomas

**Published:** 2019

**Authors:** Chitroju Bharathi, Desamala Anupama, Nallari Pratibha, Anantapur Venkateshwari

**Affiliations:** 1- Institute of Genetics and Hospital for Genetic Diseases, Osmania University, Begumpet, Hyderabad, Telangana, India; 2- Department of Gynaecology, Modern Government Maternity Hospital, Hyderabad, Telangana, India

**Keywords:** Cell proliferation, Estrogens, Leiomyoma, Myometrium, Neoplasms, Transcription factors, Uterus

## Abstract

**Background:**

Uterine leiomyomas are steroid hormone dependent myometrial neoplasms of female genital tract which appear after menarche and regress at menopause. The present study evaluated the role of ER-β gene polymorphisms (rs3020449 C/T, rs3020450 G/A, rs1271572 G/T, rs1256049 G/Aand rs4986938 G/A) in the etiology of disease.

**Methods:**

A total of 150 clinically, ultrasonographically evaluated uterine leiomyoma patients and an equal number of individuals as controls were considered for the present study. Genotype analysis was carried out by TETRA Primer Amplification Refractory Mutation System–PCR for promoter polymorphisms and PCR- RFLP method was done for exonic polymorphisms followed by agarose gel electrophoresis. The strength of the association of ER-β gene polymorphisms between controls and patients were measured by appropriate statistical methods.

**Results:**

An increased frequency of T/T genotype and T allele of rs3020449, AA genotype and A allele of rs3020450, T/T genotype and T allele of rs1271572, AA genotype and A allele of rs1256049 and A/A genotype and A allele of rs4986938 was observed in cases when compared with controls.

**Conclusion:**

The study indicates that the ER-β gene polymorphisms may act as a major genetic regulator in the etiology of uterine leiomyomas.

## Introduction

Uterine leiomyoma (UL) or fibroids are the most prevalent benign pelvic neoplasms arising from the myometrial smooth muscle layer of the uterus with a prevalence rate of 20–40% in women of reproductive age (1). They are formed from myometrial transformation of normal smooth muscle cells into monoclonal tumors. Leiomyomas are the major cause for hysterectomies in women causing significant morbidity of life (2). Therefore, it is considered to be a major socio-economic public health problem. Ovarian steroids estrogen and progesterone has been reported to be the major risk factors in the development and pathogenesis of leiomyomas. As the growth of leiomyomas is dependent on estrogens, it is evident by the fact that they occur after menarche and increase in size during pregnancy and regress after menopause (3). Two major forms of intracellular estrogen receptors ER-α and ER-β have been identified which are encoded by separate genes, ESR1 and ESR2 which are hormone nuclear receptor proteins that act as ligand-inducible transcription factors with each receptor having distinct tissue expression patterns, post-translational modifications, and cellular localization in normal and disease condition (4).

Interaction of estrogen receptor alpha (ESR1) and estrogen receptor beta (ESR2) plays a pivotal role in tunica muscularis uteri cell proliferation, differentiation, and tumorigenesis of myometrium. The chromosomal location of ESR2 gene has been frequently rearranged in uterine leiomyomata and other benign tumors which could play a major role in the etiology of leiomyomas (5). So far, many studies were conducted on the role of estrogen receptor beta (ESR2) gene polymorphisms with risk of other gynecological cancers, even though the prevalence rate of UL is very high (6, 7). However, the associated pathways responsible for the pathophysiology of the disease remain unknown. Thus, this is the first study attempted to evaluate the role of ER beta gene polymorphisms in uterine leiomyomas. Therefore, the present study hypothesized that variations in the ESR2 gene promoter and exonic regions may potentially cause alterations in its biological function and influence uterine leiomyoma risk.

The ER-β gene (ESR2) is localized on chromosome 14q23–24.1 and composed of eight exons spanning approximately 40 *kb*. ER-β like ER-α shares a similar structure and considerable homology in the DNA-binding and ligand-binding domain (8). Estradiol hormone regulates many physiological processes, including normal cell growth, development, and tissue-specific gene regulation in the reproductive tract, the central nervous, and the skeletal system by binding to ER-β receptor which regulates gene expression by binding to its cognate response element or through protein–protein interactions with other transcription factors (9).

The transcription of human ER-β gene takes place from two different promoters; they are promoter 0N and promoter 0K. Depending on the bioinformatics analysis carried out on all the polymorphisms identified in the ER-β gene, the present study has mainly focussed on three SNPs near the transcription start site of promoter 0N and two SNPs in exonic regions. The hypothesis for considering promoter region SNPs of ESR2 gene such as1271572, rs3020450 and rs3020449 was that SNPs located in this region could be able to affect transcription factor binding or may affect enhancer or repressor proteins regulating the transcription of the ESR2 gene resulting in altered ER-β protein levels. Whereas rs1256049 in exon 5 region causes a synonymous change of unknown functional significance, and rs4986938 located in the 3′-untranslated region is associated with a number of diseases, including breast cancer and endometrial cancer. The variations might interfere with some of the ER-β-proposed antiproliferative effects thus modulating estrogen effects on fibroids development (10–12).

## Methods

### Selection of cases and controls:

The present study was carried out on the blood samples collected from clinically and ultrasonographically confirmed 150 uterine leiomyoma patients and 150 control subjects referred to Government Modern Maternity Hospital, Petlaburj, Hyderabad India, during the period 2013–16. Information regarding the demographic characteristics such as age, menarche, diet, menstrual history, BMI, parity, *etc* were taken and an informed consent was obtained from all the subjects with the help of a standard proforma. The study was approved by Institutional Ethical Committee of Institute of Genetics, Osmania University, Hyderabad. The institutional ethical committee has approved all the experiments and methods which were performed in accordance with relevant guidelines and regulations.

### Inclusion criteria:

Patients diagnosed ultrasonographically with single and multiple uterine leiomyomas in range of age from 25–45 years were included in the present study.

### Exclusion criteria:

Patients diagnosed with any other reproductive diseases and pregnant women with leiomyoma were excluded from the study.

Age matched healthy women were randomly selected from population as control subjects and were clinically and ultrasonographically confirmed without prior history of reproductive abnormalities for comparative analysis.

### DNA extraction:

The venous blood (5 *ml*) was drawn from each individual in vacutainers containing EDTA and stored at 4*°C*. Genomic DNA was extracted from the peripheral blood using salting out method described by Lahiri et al. (1991) (13) and stored in TE buffer at −20*°C* until further use.

### Determination of ER-β gene promoter polymorphisms:

Allele-Specific Tetra-Primer amplification was performed on the genomic DNA using a Tetra-Primer ARMS PCR for genotyping the ER- β (rs3020449 C/T, rs3020450 G/A and rs1271572 G/T) gene promoter polymorphisms. Each PCR reaction was carried out in a total volume of 10 *μl*, containing 40 *ng* of template DNA, 10 *pmol* of each inner primer, 1 *pmol* of each outer primer, 0.2 *mM* dNTP, and 1 unit Taq DNA polymerase (Bangalore Genei, India). The primers used for PCR reaction are listed in table 1. The PCR cycling conditions for the detection of rs3020449 and rs3020450 were as follows: 95*°C* for 5 *min*, then 24 cycles of 95*°C* for 1 *min*, 56*°C* for 1 *min*, and 72*°C* for 1 *min*, followed by 72*°C* for 5 *min*. For the detection of rs1271572, the following cycling conditions were utilized: 94*°C* for 5 *min*, 25 cycles of 94*°C* for 30 *s*, 52*°C* for 1 *min* and 72*°C* for 1 *min*, followed by 72*°C* for 5 *min*. PCR products were mixed with 3 *μl* of loading buffer and analyzed using 2.5% agarose gel electrophoresis. Allele-Specific PCR product sizes were 353/250 *bp* (C/T) for SNP rs3020449, 419/209 *bp* (G/A) for rs3020450 and 276/133 *bp* (G/T) for rs1271572.

**Table 1.** PCR primers used for genotype analysis of ER-β gene

**Polymorphism**	**Primer**	**Sequence**
**rs3020449(C/T)**	Forward outer primer	5′ CCCAGATGGCTTCAGTG 3′
	Reverse outer primer	5′ CCGTCCTGTCCTTAAAAGTA 3′
	Forward inner primer	5′ CAAGGAAATTTTAGCAAATCC 3′
	Reverse inner primer	5′ CCTTTTTACATATTGTTAGGTTA 3′
**rs3020450(G/A)**	Forward outer primer	5′ TCCGTTCACTGTCTTCTCTAC 3′
	Reverse outer primer	5′ TGGGGTCTCTTCTGAATTAC 3′
	Forward inner primer	5′ CCTTGTGTTCTCTGTTCTCTCCA 3′
	Reverse inner primer	5′ GAAGAGAGCCCAGGATTTCTAC 3′
**rs1271572(G/T)**	Forward outer prime	5′ CCCCTCGTCTTCCTCTATTA 3′
	Reverse outer primer	5′ ACCGGGGAGACCTGTG 3′
	Forward inner primer	5′ GATGTGACACTGGGGGGG 3′
	Reverse inner primer	5′ CCACAGGCCATTGTGAGAA 3′
**rs4986938 (G/A)**	Forward primer	5′ GAGGAGACGGACCAAAGCCAC 3′
	Reverse Primer	5′ GCCATTGGTGTTGGATGCATG 3′
**rs1256049(G/A)**	Forward primer	5′ CTGCCACCCTATCTGTATCTTTTCCTATTCTCC 3′
	Reverse primer	5′ TCTTTCTCTGCCACCCTGGCGTCGATTATCTGA 3′

### Determination of ER-β +1730 and 1820 gene polymorphisms:

Molecular analysis of the ER-β gene rs4986938 G/A (+1730) and rs1256049 G/A (1820) polymorphism was performed using PCR with appropriate primers given in table 1 followed by RFLP. The PCR reaction was carried out in a final volume of 25 *μl*, containing 1X buffer, 2.5 *mM* MgCl2, 0.1 *mM* of each dNTP, 50 *nM* of each primer, 1 *U* of Taq polymerase (Bangalore Gene, India) and 40 *ng* of DNA. Amplification of +1730 G/A was performed with an initial denaturation step at 95*°C* for 5 *min*, followed by 33 cycles at 95*°C* for 1 *min*, annealing at 49*°C* for 1 *min*, and extension at 72*°C* for 1 *min* and a final extension step at 72*°C* for 1 *min*, and for the amplification of 1820 G/A polymorphism includes the cycling conditions of initial denaturation step at 95*°C* for 5 *min*, followed by 33 cycles at 95*°C* for 1 *min*, annealing at 54*°C* for 1 *min*, and extension at 72*°C* for 1 *min* and a final extension step at 72*°C* for 1 *min*. Next, the products were separated in a 3% agarose gel stained with ethidium bromide and visualized under UV light. The PCR products were restriction digested using AluI restriction enzyme for the site (+1730) which produces bands of sizes that presented a 307 *bp* in the normal ER-β sequence (GG), three separate bands of 307, 240, and 67 *bp* in the heterozygous polymorphism (GA) and the homozygous polymorphism (AA) produced two separate bands of sizes 240 and 67 *bp*. The restriction digestion of 1820 G/A exon 5 polymorphism with RSAI enzyme produced bands of sizes 150 *bp* in the normal ERβ sequence (GG), three separate bands of 150, 125, and 37 *bp* in the heterozygous polymorphism (GA) and two separate bands of 125 and 37 *bp*, respectively in the homozygous polymorphism (AA).

### Statistical analysis:

Genotypic distribution and allele frequencies of case and control group were analyzed by chi-square test to assess the Hardy-Weinberg equilibrium. Major and minor alleles of each SNP were represented as 1 and 2, respectively. Fischer exact test (2-tailed) was performed to determine the difference in the distribution of genotypes and alleles in patients and control group. p-value <0.05 is considered significant. To measure the strength of association between ER-β gene promoter (rs3020449, rs3020450, rs1271572) and exon polymorphisms (rs4986938 G/A+1730 and rs1256049 G/A1820) with uterine leiomyomas, odds ratios (OR) and their 95% confidence intervals (CI) were calculated. Statistical analysis between groups was determined by *χ*^2^ test using SNPstat and openEpi softwares. The coefficient (D’) of pairwise linkage disequilibrium (LD), the non-random association between the SNPs was calculated using the haploview version 4·2.

## Results

A total of 150 uterine leiomyoma patients and an equal number of controls without any abnormal reproductive history were included in the present study. The demographic characteristics of patients and controls presented in table 2 revealed a significant difference with respect to age (p=<0.001), age of menarche (p=<0.001), parity (p=<0.001), menorrhea (p=<0.001), diet (p=<0.001) and BMI (0.005). The p-values were further adjusted following the Multiple Logistic Regression. The analysis revealed adjusted p-values to be significant for all the variables.

**Table 2.** Demographic features of uterine leiomyoma patients and control subjects

	**Controls n (%)**	**Uterine fibroids n (%)**	**χ^2^**	**OR(95%CI)**	**p-value**	**Adjusted p-value MLR**
**Age (years)**						
≤30	66(44.00)	35(23.33)				
>30	84(56.00)	115 (76.66)	13.43	2.58(1.57–4.24)	<0.001	<0.001
BMI *						
Normal weight	80(53.33)	55 (36.66)				
Over weight	70(46.66)	95 (63.33)	7.75	1.97(1.24–3.13)	0.005	<0.001
**Menarche (years)**						
10–12	65(43.33)	96 (64.00)				
13–15	85(56.66)	54 (36.00)	12.06	0.43(0.27–0.68)	<0.001	<0.001
**Parity (n)**						
Parous	109(72.66)	65 (43.33)				
Nulliparous	41(27.33)	85 (56.66)	25.30	3.47(2.14–5.63)	<0.001	<0.001
**Diet**						
Vegetarian	48(32.00)	16 (10.66)				
Non-Vegetarian	102(68.00)	134 (89.33)	19.09	3.94(2.11–7.33)	<0.001	<0.001
**Menorrhea**						
Eumenorrhea	105(70.00)	57 (38.00)				
Dysmenorrhea	45(30.00)	93 (62.00)	29.64	3.80(2.35–6.15)	<0.001	<0.001

* BMI = weight (*kg*)/height ^2^ (*m*^2^)

### Age:

As the age increases, the prevalence of fibroids increases during late reproductive years and this is due to 20–30 years of stimulation by pre-menopausal hormonal factors like estrogens and progesterones that act as modulators in the development of fibroids (14).

### BMI:

Women with higher BMI were found to have increased risk of developing fibroids due to increase in biologically available estrogens; it occurs in two ways as the adipose tissue converts the adrenal and ovarian androgens into estrogens and several obesity related mechanisms lead to decreased synthesis of sex hormone binding globulin thus ultimately resulting in increased growth and prevalence of leiomyomas by estrogens.

### Age at Menarche:

Early age at menarche might increase the number of myometrial cell divisions during the reproductive years, leading to increased risk for mutations in genes controlling myometrial proliferation thus, increasing the risk of leiomyoma.

### Parity:

Increase in parity reduces the risk of leiomyomas and this could be due to a remodeling process of the extracellular matrix (ECM) and a specific expression of receptors for peptide and steroids hormones induced by pregnancy and parturition (15). Pregnancy reduces the time of exposure to unopposed estrogens, whereas nulliparity or reduced fertility may be associated with anovulatory cycles characterized by long-term unopposed estrogens.

### Diet:

Several findings reported that the modulation of diet can influence estrogen metabolism in premenopausal women which may influence the risk of fibroids. Vegetarians consumed less fat and more dietary fiber than did the non-vegetarians which reduces serum estrogen levels, probably by altering the fecal flora and reducing the enterohepatic circulation of estrogens (16).

### Menorrhea:

Most women suffering from uterine fibroids encounter severe painful periods as the size and position of myomas cause pressure in the pelvic region which could be responsible for dysmenorrhea in fibroid patients and normal healthy women even though they have pain less severe than patients with fibroids.

The allelic and genotypic frequencies of the five studied ER-β polymorphisms in control and patient subjects are shown in tables 3 and 4.

**Table 3.** Distribution of allele frequencies of ER-β gene polymorphisms in patients with uterine leiomyoma and controls

**Allele frequency**	**Controls n (%)**	**Cases n (%)**	**χ^2^**	**OR(95%CI)**	**p-value**
**rs3020449**					
C	206 (69.00)	110 (37.00)			
T	94 (31.00)	190 (63.00)	60.34	3.78 (2.69–5.31)	<0.001
**rs3020450**					
G	205 (68.00)	127 (42.00)			
A	95 (32.00)	173 (58.00)	39.98	2.93 (2.10–4.10)	<0.001
**rs1271572**					
G	116 (39.00)	29 (10.00)			
T	184 (61.00)	271 (90.00)	67.26	5.89 (3.76–9.22)	<0.001
**rs1256049**					
G	242 (81.00)	195 (65.00)			
A	58 (19.00)	105 (35.00)	17.82	2.24 (1.54–3.25)	<0.001
**rs4986938**					
G	221 (74.00)	122 (41.00)			
A	79 (26.00)	178 (59.00)	65.37	4.08 (2.89–5.76)	<0.001

**Table 4.** Distribution of genotype frequencies of ER-β gene polymorphisms in patients with uterine leiomyoma and controls

**Genotype frequency**	**Controls n (%)**	**Cases n (%)**	χ^**2**^	**OR (95% CI)**	**p-value**
**rs3020449**					
**Co-dominant**					
C/C	81(54.00)	18(12.00)			
C/T	44(29.30)	74(49.30)	41.91	7.56(4.02–14.25)	<0.001
T/T	25(16.70)	58(38.70)	47.51	10.44(5.21–20.88)	<0.001
**Dominant**					
C/C	81(54.00)	18(12.00)			
C/T+T/T	69(46.00)	132(88.00)	57.95	8.60(4.78–15.50)	<0.001
**Recessive**					
C/T+C/C	125(83.30)	92(61.30)			
T/T	25(16.70)	58(38.70)	17.06	3.15(1.83–5.41)	<0.001
**Overdominant**					
C/C+T/T	106(70.70)	76(50.70)			
C/T	44(29.30)	74(49.30)	11.75	2.34(1.45–3.77)	<0.001

**rs3020450**					
**Co-dominant**					
G/G	79(52.70)	27(18.00)			
G/A	47(31.30)	73(48.70)	27.11	4.54(2.56–8.03)	<0.001
A/A	24(16.00)	50(33.30)	29.85	6.09(3.16–11.72)	<0.001
**Dominant**					
G/G	79(52.70)	27(18.00)			
G/A+A/A	71(47.30)	123(82.00)	37.94	5.06(2.99–8.57)	<0.001
**Recessive**					
G/A+G/G	126(84.00)	100(66.70)			
A/A	24(16.00)	50(33.30)	11.21	2.62(1.51–4.56)	<0.001
**Overdominant**					
G/G+A/A	103(68.70)	77(51.30)			
G/A	47(31.30)	73(48.70)	8.68	2.07(1.29–3.32)	0.003

**rs1271572**					
**Co-dominant**					
G/G	00.00	00.00			
G/T	116(77.30)	29(19.30)			
T/T	34(22.70)	121(80.70)	98.72	14.24(8.15–24.85)	<0.001
**Dominant**					
G/G	00.00	00.00			
G/T+T/T	150(100.00)	150(100.00)	------	------	------
**Recessive**					
G/T+G/G	116(77.30)	29(19.30)			
T/T	34(22.70)	121(80.70)	98.72	14.24(8.15–24.85)	<0.001
**Overdominant**					
G/G+T/T	34(22.70)	121(80.70)			
G/T	116(77.30)	29(19.30)	98.72	0.07(0.04–0.12)	<0.001

**rs1256049**					
**Co-dominant**					
G/G	98(65.30)	72(48.00)			
G/A	46(30.70)	51(34.00)	2.20	1.50(0.91–2.49)	0.137
A/A	06(4.00)	27(18.00)	15.68	6.12(2.40–15.61)	<0.001
**Dominant**					
G/G	98(65.30)	72(48.00)			
G/A+A/A	52(34.70)	78(52.00)	8.48	2.04(1.28–3.24)	0.003
**Recessive**					
G/A+G/G	144(96.00)	123(82.00)			
A/A	06(4.00)	27(18.00)	13.62	5.26(2.10–13.18)	<0.001
**Overdominant**					
G/G+A/A	104(69.30)	99(66.00)			
G/A	46(30.70)	51(34.00)	0.24	1.16(0.71–1.89)	0.621
**rs4986938**					
**Co-dominant**					
**G/G**	83(55.3)	29(19.30)			
**G/A**	55(36.7)	64(42.70)	17.52	3.33(1.91–5.80)	<0.001
**A/A**	12(8)	57(38.00)	52.82	13.59(6.40–28.85)	<0.001
**Dominant**					
G/G	83(55.3)	29(19.30)			
G/A+A/A	67(44.7)	121(80.70)	40.02	5.16(3.08–8.67)	<0.001
**Recessive**					
G/A+G/G	138(92)	93(62.00)			
A/A	12(8)	57(38.00)	36.44	7.04(3.58–13.85)	<0.001
**Overdominant**					
G/G+A/A	95(63.3)	86(57.30)			
G/A	55(36.7)	64(42.7)	0.89	1.28(0.80–2.04)	0.345

### ER-β rs3020449 C/T polymorphism:

The frequencies of genotype CC, CT and TT were 54%, 29.3% and 16.7% in controls and 12%, 49.3% and 38.7% in patients with uterine leiomyoma, respectively. The C and T allele frequency among control group was 69% and 31% and in patient group was 37% and 63%, respectively. There is a statistically significant difference in the distribution of allelic and genotypic frequencies. The T allele shows a threefold increased risk, and an increase in ten-fold risk was observed with respect to TT genotype in cases when compared to their respective controls.

### ER-β rs3020450 G/A polymorphism:

The frequencies of genotype GG, GA and AA were 52.7%, 31.3% and 16% in controls and 18%, 48.7% and 33.3% in patients with uterine leiomyoma, respectively. The frequency of G and A alleles among control group was 68% and 32% and in patient group was 42% and 58%, respectively and a significant association of A allele with two-fold increased risk was observed. The AA genotype revealed an increased risk of six-fold in cases when compared to their controls.

### ER-β rs1271572 G/T polymorphism:

The frequencies of genotype GG, GT and TT were 0% 77.3% and 22.7% in controls and 0%, 19.3% and 80.7% in patients with uterine leiomyoma, respectively. The homozygous wild genotype GG was not observed in cases and controls. The frequency of G and T alleles among control group was 39% and 61% and in patient group was 10% and 90%, respectively. However, a significant association of “T” allele with a five-fold increased risk was observed in cases when compared to control subjects.

### ER-β rs1256049 G/A polymorphism:

The frequencies of genotype GG, GA and AA were 65.3%, 30.7% and 04% in controls and 48%, 34% and 18% in patients with uterine leiomyoma, respectively. The frequency of G and A alleles among control group was 81% and 19% and in patient group was 65% and 35%, respectively. A two-fold increased risk was observed with A allele and a significant association with respect to AA genotype with a six-fold increased risk was observed in cases when compared to their respective controls.

### ER-β rs4986938 G/A polymorphism:

The frequencies of genotype GG, GA and AA were 55.3%, 36.7% and 08% in controls and 19.3%, 42.7% and 38% in patients with uterine leiomyoma, respectively. The frequency of G and A alleles among control group was 74% and 26% and in patient group was 41% and 59%, respectively, with a four-fold increased risk of A allele. An increased risk of thirteen-fold was observed with respect to AA genotype in cases when compared to their controls, respectively.

### Linkage disequilibrium analysis:

**Figure 1.**
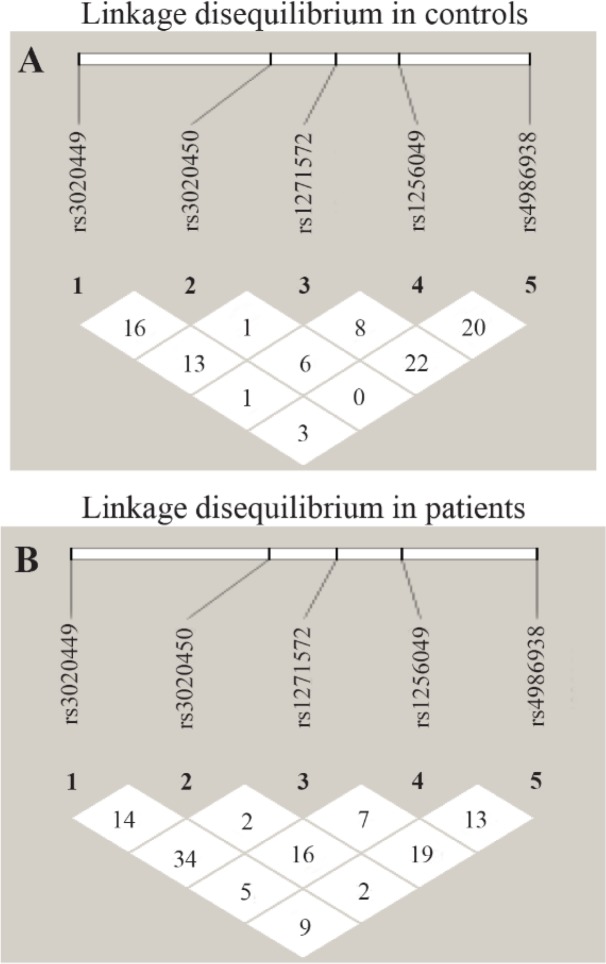
(a) and (b). Linkage disequilibrium analysis in controls and patients

Linkage disequilibrium (LD) analysis, defined by the delta coefficient (D′), was estimated in both case and control groups for the 5 SNPs of ER-β rs3020449C/T, rs3020450 G/A, rs1271572G/T in rs1256049G/A and rs4986938G/A. LD analysis revealed no linkage in the control and uterine fibroid patient group as presented in figure 1 (a) and (b) with respect to the five SNPs studied in the ER-β gene.

## Discussion

Estrogen produced in the ovaries controls the secretion of pituitary gonadotropins and is a key intra ovarian modulator, contributes to oocyte maturation, fertilization, and embryo quality. Moreover, estrogen plays a crucial role in embryonic and fetal development, influencing secondary sexual characteristics, reproductive cycle, fertility and maintenance of pregnancy (17).

Estrogens exert their effects on target cells through the activation of estrogen receptors which are classified as nuclear estrogen receptors (ERs) and membrane bound receptors (mERs) (18). The progression of cell proliferation in the G1 phase of the cell cycle is stimulated by estrogen in various target tissues, like uterus and breast. Evidence has shown that the concentration of estrogen in leiomyoma tissue is higher compared to the normal myometrium and the growth of tumor is influenced by both serum and tumor tissue estrogen levels indicating their sensitivity (19). ER-β mRNA was reported to be expressed at higher levels in leiomyomas when compared with matched myometrium (20). ER-β acts as a negative regulator of ER-α thereby exerting antiproliferative and apoptotic effect in different tissues as leiomyomas markedly regress in the postmenopausal state. Thus, it is tempting to speculate that ER-β could play a major role in the pathobiology of uterine leiomyomas.

Several polymorphisms are identified in the ESR1 gene with their conflicting results in the susceptibility of uterine fibroids; however no study examining the association of ESR2 gene polymorphisms with susceptibility to uterine fibroids reported since, discovery of this gene and its function (21–24). A number of promoter, exonic and intronic polymorphisms in the ER-β gene have been identified, showing their direct biological significance and association with human diseases.

In the present case control study, a total of five SNPs three in the promoter region given (rs3020449, rs3020450, rs1271572) and two in the exonic region (rs1256049, rs4986938) of the ER-β gene were selected (http://www.ncbi.nlm.nih.gov/snp) and investigated as candidates for conferring variations in all patients with uterine fibroids compared to healthy individuals. Our results showed that there is a significant association of polymorphisms in cases compared with their respective control groups, thereby indicating the role of ER-β gene as an independent prognostic risk factor in the etiology of uterine fibroids.

Very few studies were reported from different populations to determine the association of different ER-β gene polymorphisms in uterine fibroids and other diseases at the genotype and the haplotype level. The present study revealed a significant association of ER-β rs4986938 (+1730 G/A), rs1256049 (1082 G/A) polymorphism and uterine fibroids. In contrary, earlier studies by Massart et al. (2003) in Caucasian population reported no association between +1730 G/A polymorphism and uterine fibroids (25). A study reported by Wang et al. (2004) in Japanese population on 1082 G/A polymorphism did not show any significant association with endometriosis (26). Our study is supported by other studies by Seleem et al. (2014) wherein a significant association of +1730 G/A polymorphism was observed with the risk of endometriosis (27). Sundarrajan et al. (2001) observed an association of ER-β +1730 G/A and 1082 G/A SNPs in ovulatory dysfunctions (28). Wang et al. (2004) suggested the association of ER-β +1730 G/A polymorphism in endometriosis in Japanese population (26). Abbas et al. (2010) reported a significant association of +1730 G/A polymorphism in women with post-menopausal breast cancer risk (29). Zulli et al. (2010) reported the association of +1730 G/A with risk of infertility and endometriosis associated infertility (17). A case control study done in North Indian population by Ghosh et al. (2012) revealed no significant associations for rs1271572 and rs1256049 at genotypic and allelic levels and no linkage disequilibrium was observed between these two SNPs (30). A study by Zhai et al. (2009) found no association between ESR2 (rs1256049 and rs928554) polymorphisms and uterine leiomyoma (5).

The present study has revealed a significant association of three selected ER-β promoter polymorphisms (rs3020449, rs3020450 and rs1271572) between cases and control group in contrary to the study done by Lattrich et al. (2014) in endometrial cancer revealed no significant association of rs3020450 and rs3020449 polymorphism (31). Treeck et al. (2009) found no significant association of rs3020450 and rs3020449 promoter polymorphisms with breast cancer risk (32). The study by Li Chen et al. (2013) in Chinese population reported no association of rs3020450 and rs3020449 polymorphism but found a significant association of rs1271572 polymorphism in breast cancer patients (12). Abbas et al. (2010) reported a significant association of rs1271572 G/T polymorphism in women with post-menopausal breast cancer risk (29).

The LD analysis of five SNPs revealed no linkage disequilibrium in either controls or patient subjects. A study by Fischer et al. (2010) in ER-β gene (rs2987983, rs3020450 and rs3020449) promoter polymorphisms and their haplotype analysis in uterine fibroids did not reveal any significant association and linkage disequilibrium (21), since in the absence of LD, no haplotypes were constructed in the present study. There is presently no evidence that ESR2 is a major genetic determinant of leiomyoms risk. However, the genetic contribution of ESR2 to leiomyoma risk needs to be further explored. Based on our findings, it could be hypothesized that the polymorphisms in the promoter 0N may lead to altered protein expression or its function (12). Evidence by Borahay et al. (2015) have supported that the aberrant estrogen receptor signalling contributes to leiomyoma growth and development (18). Thus, the present study mainly focussed on promoter 0N polymorphisms, wherein the genotypic analysis of these SNPs revealed a significant association with the risk of uterine fibroids.

To the best of our knowledge, this is the first study reporting the correlation between five polymorphisms of ER-β gene and uterine fibroids from Telangana state of South India.

## Conclusion

In conclusion, the present study reports a significant association of the genotype and allele frequencies of the five studied polymorphisms (rs 3020449C/T, rs3020450G/A, rs1271572G/T in rs1256049G/A and rs4986938G/A) in uterine fibroid patients when compared with their respective controls. The study revealed no linkage disequilibrium of the five SNPs in either control or patient group ultimately; the present case-control study clearly suggests that the five SNPs of ER-β gene act individually as prognostic markers in the etiology of UL. Our data further encourages to continue the investigation of the significance of ER-β SNPs in combination with other gene polymorphisms, and their influence on the risk of uterine fibroids.
